# Epidemiology, Drug Susceptibility, and Clinical Risk Factors in Patients With Invasive Aspergillosis

**DOI:** 10.3389/fpubh.2022.835092

**Published:** 2022-04-15

**Authors:** Yuerong Wang, Luwen Zhang, Longrong Zhou, Min Zhang, Yuanhong Xu

**Affiliations:** Department of Clinical Laboratory, The First Affiliated Hospital of Anhui Medical University, Hefei, China

**Keywords:** *Aspergillus* spp., invasive aspergillosis, antifungal sensitivity, risk factors, azole resistance

## Abstract

**Background:**

This study aimed to investigate the *Aspergillus* species distribution, antifungal sensitivities, clinical characteristics, and risk factors of patients with invasive aspergillosis (IA) in a tertiary teaching hospital in Anhui Province.

**Methods:**

In the present study, 156 *Aspergillus* isolates were collected from patients admitted to a 2,800-bed comprehensive hospital between January 2019 and April 2021. The epidemiology of *Aspergillus* species was well-examined, and its antifungal susceptibility was specifically measured by the microbroth dilution method. The risk factors of patients with IA were documented and analyzed intensively. In addition, gene sequencing was employed to determine gene mutations of cytochrome P450 14-α sterol demethylase*-Aspergillus* (*cyp51A*) associated with azole resistance among *Aspergillus fumigatus*.

**Results:**

The *Aspergillus* species distribution was dominated by *A. fumigatus* (56.41%), *Aspergillus flavus* (20.51%), and *Aspergillus niger* (15.38%) locally. In particular, all *Aspergillus* species showed very low minimum inhibitory concentrations (MICs, ≤ 0.5 μg/ml) for azoles and echinocandins, slightly high MICs (1.66–2.91 μg/ml) for amphotericin B, and exceptionally high MICs (>64 μg/ml) for flucytosine. Azole-resistant rate of *Aspergillus* species in this local region reached up to 5.79%. Correlation analyses of multiple antifungals indicate a significant MIC relevance between isavuconazole and voriconazole (Pearson correlation coefficient was 0.81, *P* < 0.0001). The clinical risk factors for patients with IA were found primarily to be pulmonary diseases (*P* = 0.007) and patients' age (*P* < 0.001). Notably, three mutant loci (TR46/Y121F/T289A) of the *cyp51A* gene were identified in azole-resistant *A. fumigatus*.

**Conclusions:**

The *Aspergillus* species emerged increasingly, of which *A. fumigatus* and *A. flavus* remained the main pathogens for invasive *Aspergillus* infections in the local region. The vast majority of *Aspergillus* species exhibited good susceptibility to all the antifungals, except flucytosine. The local occurrence of azole-resistant *Aspergillus* species grew gradually and needed monitoring in time. Pulmonary diseases and age were likely considered as highly associated risk factors for IA. To our knowledge, the clinically isolated azole-resistant *A. fumigatus* with TR46/Y121F/T289A mutations identified here were rarely reported in the area of China.

## Introduction

Invasive aspergillosis (IA) is clinically one of the most serious invasive fungal infections, with high morbidity, mortality, and costs of care (1). Over 200,000 cases of IA are reported to be infected with *Aspergillus* species each year (2). *Aspergillus fumigatus* is the most frequently encountered *Aspergillus* spp. that causes IA and allergic diseases, although other species, such as *Aspergillus flavus, Aspergillus niger, Aspergillus terreus, Aspergillus versicolor*, and *Aspergillus nidulans*, can also induce diseases (3, 4). Immunocompromised patients and those with underlying lung diseases are susceptible to IA, ranging from acute IA to chronic pulmonary aspergillosis (CPA) (5, 6). However, early diagnosis of IA is difficult and its misdiagnosis happens readily since *Aspergillus* infections seldom have characteristic manifestations and the specific pathogenic agents regularly take a long time to be detected (7).

Current antifungal resistance situations are of serious concern (8–10), although azole antifungals prove critical in the treatment of IA, including voriconazole, isavuconazole, posaconazole, itraconazole, *etc*. for first-line or remedial regimens (11). As the incidence of aspergillosis is increasing year by year, the resistance rate of *Aspergillus* spp. to azoles also gradually rises, and particularly the resistance of *A. fumigatus* to azoles in different countries/regions varies diversely. To date, studies have identified alterations in cytochrome P450 sterol 14α-demethylase (CYP51), a target protein within many azole-resistant *A. fumigatus*, which likely impair antifungal binding ability as a result of amino acid substitutions due to single nucleotide polymorphisms of the gene encoding CYP51*-Aspergillus* (CYP51A) protein (12). Therefore, clinical awareness and assessment of prevailing strains, epidemiological characteristics, and drug sensitivity profiles of invasive *Aspergillus* infections in local regions are key to early clinical treatment and improvement of clinical outcomes of patients.

In this study, we investigated the species distribution and drug sensitivities of *Aspergillus* spp. from patients with IA and their clinical characteristics and risk factors in combination with corresponding clinical data in the local Anhui Province of China. Notably, the gene encoding CYP51A in azole-resistant *A. fumigatus* was sequenced to potentially explore a possible molecular reason for the antifungal resistance.

## Materials and Methods

### Patient Data Collection

We routinely harvested fungal isolates from patients who developed IA in a tertiary hospital containing a comprehensive 2,800-bed facility affiliated to a medical university of Anhui Province in central China between January 2019 and April 2021. Demographic information and clinical data were retrieved from a computerized hospital data system. Specimens from both sterile and non-sterile body sites were carefully collected, among which samples from the conventionally non-sterile body sites were obtained aseptically by vesicopuncture, or from deep lower respiratory tracts of patients with IA primarily by bronchofiberscopy, for direct examination under microscopy and mycological cultures. All the cases were categorized as proven, probable, or possible IA according to the revised definitions of invasive fungal disease (IFD) from the European Organization for the Research and Treatment of Cancer/Mycoses Study Group (EORTC/MSG) consensus group (13), and based on the Chinese expert consensus statement issued by the Chinese Medical Association (14, 15). Briefly, the proven IA requires *Aspergillus* identification in pathological tissues or fungal cultures of specimens obtained by aseptic procedures from sterile normal or clinically/radiologically abnormal sites consistent with infectious disease processes. The probable IA is mainly clinically probable invasive pulmonary aspergillosis, which was diagnosed by host factors, clinical features, and mycological evidence. For the host factors, our subjects were principally cancer and/or organ transplant patients with long-term use of hormones or immunosuppressants within the past 60–90 days. The clinical features revealed that part of the patients presented the following patterns on CT images: dense, well-circumscribed lesions with/without halo signs, air crescent signs, cavities, wedge-shaped, or segmental/lobar consolidations. In other patients, tracheobronchial ulcers, nodules, pseudo-membranes, plaques, or eschars were visible *via* bronchoscopy. For the mycological evidence, *Aspergillus* spp. were recovered by culture and/or microscopic detection of fungal elements from deep lower respiratory tracts, bronchoalveolar lavage fluid (BALF), bronchial brush biopsy, or aspirates. Only one strain of *Aspergillus* spp. was isolated from colorectal contents of a patient with Crohn's disease that was considered as the possible IA, with the fact that the absence of fungi and blood culture-negative results were observed after antifungal treatment. The present study ruled out the repeatedly isolated fungal species from the same patients. Multiple risk factors were collected for the analysis of IA incidence in the local region.

### Microorganism Identification

All clinical specimens were inoculated to plates of Sabouraud Dextrose agar and CHROMagar *Candida* agar(Hefei Tianda Biotech Co., Ltd., Anhui, China) for culture at 35°C for 48 h, except blood samples which were processed using a BacT/ALERT 3D automated microbial detection system (BacT/ALERT 3D, bioMérieux, Marcy l'Étoile, France). *Aspergillus* spp. were identified by morphological identifications combined with matrix-assisted laser desorption ionization-time of flight mass spectrometry (MALDI-TOF MS) on a Vitek MS system (bioMérieux, Marcy l'Étoile, France), and DNA sequencing (Sangon Biotech Co., Ltd., Shanghai, China) if ambiguity with MS occurs. Sample preparations for MS analysis were conducted as described previously (16). It was considered acceptable for the identification results when the MS confidence value was 99.9%. The DNA sequencing for *Aspergillus* species identification was conducted using two universal fungal primers for internal transcribed spacer (ITS1-forward: 5′-TCC GTA GGT GAA CCT GCG G-3′, ITS4-reverse: 5′-TCC TCC GCT TAT TGA TAT GC-3′) (17).

### Antifungal Susceptibility Testing

The antifungal susceptibility test for *Aspergillus* spp. *via* Sensititre YeastOne (SYO) kit (Zhuhai DL Biotech Co., Ltd., Shenzhen, China) was carried out in accordance with the manufacturer's instructions. Briefly, after being cultured on potato dextrose agar at 35°C for 7 days, conidia were harvested using sterile saline containing Tween-20. The conidial inoculum suspension was prepared at a turbidity of 0.5 McFarland units for the assay. With 48-h of culturing (except echinocandins which were cultured for 24-h), the antifungal drug sensitivity profiles of these inocula were finally available according to the interpretations of the kit instructions. For those initially tested as resistant isolates, we repeated the assays to confirm the testing results. The present study did not involve fluconazole that is not active against *Aspergillus* spp. due to its inherent resistance. Quality control strains included *Candida krusei* ATCC6258 and *Candida parapsilosis* ATCC22019. Specifically, the minimum inhibitory concentrations (MICs) for amphotericin B (AMB), flucytosine (FC), and four azoles, including isavuconazole (ISA), voriconazole (VRC), posaconazole (POS), and itraconazole (ITR), and the minimum effective concentrations (MECs) for micafungin (MIF) and caspofungin (CAS) were determined for all the *Aspergillus* inocula. The interpretative criteria for susceptibility and breakpoints for antifungal drugs were referenced to the European Committee on Antibiotic Susceptibility Testing (EUCAST), and the Clinical and Laboratory Standards Institute (CLSI) M38-A3 microbroth dilution method.

### DNA Sequencing of Azole-Resistance Related Gene

The promoter and coding regions of the *cyp51A* gene were amplified by the PCR and separately sequenced using previously published primers (18). Briefly, the primers for the *cyp51A* promoter region were the following: PA7F 5′-TCATATGTTGCTCAGCGG-3′ and PA5R 5′-TCTCTGCACGCAAAGAAGAAC-3′ (19). PCR conditions included as follows: 95°C for 10 min, 35 cycles of 94°C for 1 min, 43°C for 45 s, and 72°C for 1 min, followed by a final extension at 72°C for 7 min. The primers for the coding region of cyp*51A* were the following: P450A1 5′-ATGGTGCCGATGCTATGG-3′ and P450A2 5′-CTGTCTCACTTGGATGTG-3′ (20). PCR conditions were as follows: 95°C for 5 min, 35 cycles of 94°C for 3 min, 47.4°C for 45 s, and 72°C for 2 min, with a final extension at 72°C for 7 min. The PCR products were semi-quantified by agarose gel electrophoresis and used as templates for sequencing (Sangon Biotech Co., Ltd., Shanghai, China). The resultant fragment sequences of promoter and coding regions of the *cyp51A* gene were aligned with the ones deposited in GenBank (Registration number: AF338659) (19) and were thoroughly analyzed using the Meg Align software (DNA Star, Inc., Lasergene, Madison, WI, USA).

### Statistical Analysis

The data of geometric mean of MICs, MIC_50_, MIC_90_, and Pearson χ^2^ test were processed with IBM SPSS Statistics 21 (SPSS Inc., Chicago, IL, USA), and *P* < 0.05 was considered significantly different. Correlation analyses of MICs for azoles were performed using GraphPad Prism software version 5 (GraphPad Software Inc., San Diego, CA, USA).

### Ethics Statement

The protocols of the study were approved by and carried out following the recommendations of the Life Ethics Committee of Anhui Medical University. All subjects gave their written informed consents as per the Declaration of Helsinki.

## Results

### Clinical Distribution Characteristics of Invasive *Aspergillus* Infections

**Figure 1:**
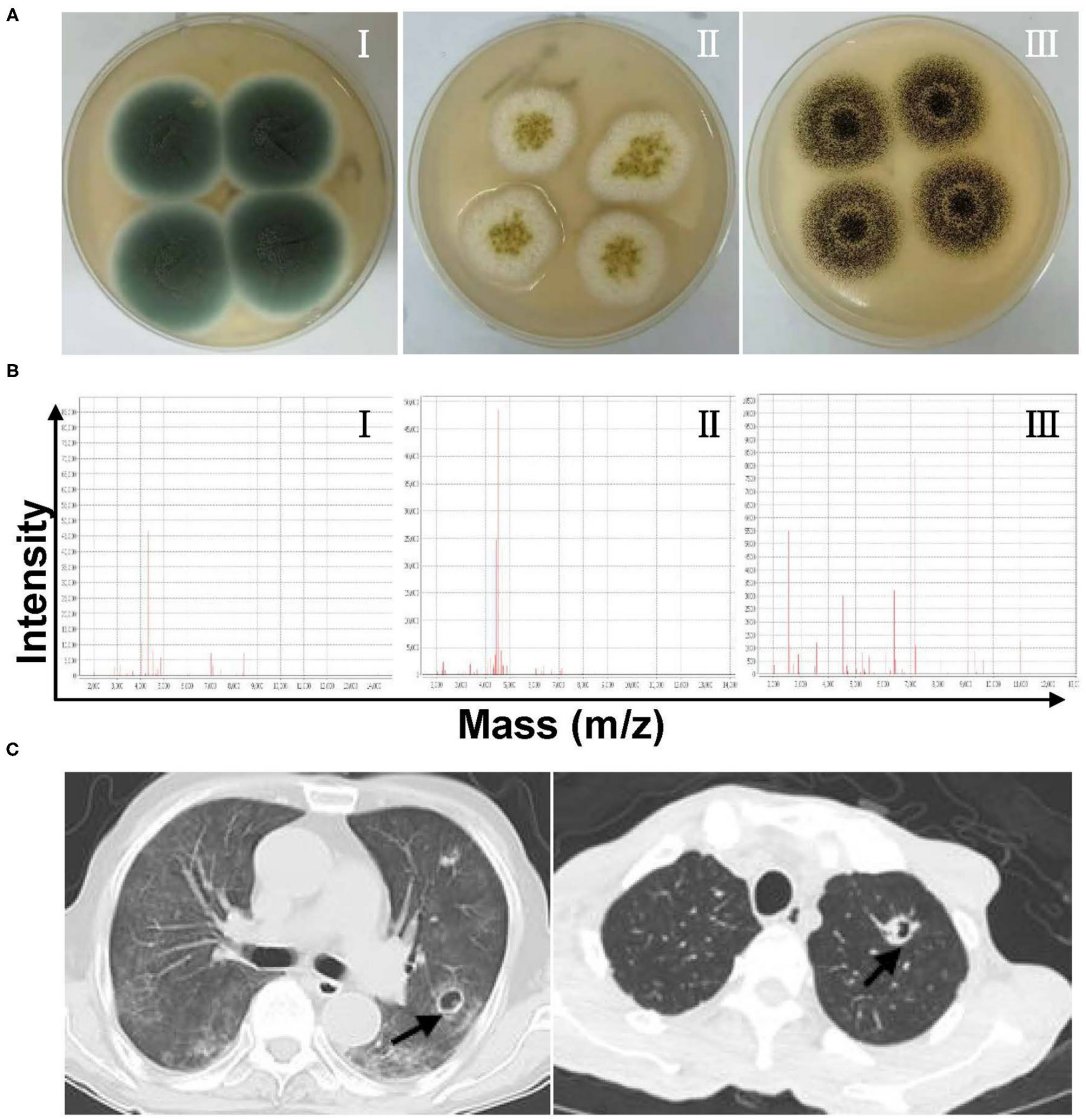
**(A)** Representative culturing macroscopic results of *Aspergillus* spp. (Sabouraud Dextrose agar medium). I–III: *Aspergillus fumigatus, Aspergillus flavus*, and *Aspergillus niger*. **(B)** Representative identification information of *Aspergillus* spp. by matrix-assisted laser desorption ionization-time of flight mass spectrometry (MALDI-TOF MS). I–III: *A. fumigatus, A. flavus*, and *A. niger*. **(C)** Chest CT imaging features of patients with invasive pulmonary aspergillosis.

During the study period, 156 *Aspergillus* isolates were collected and identified to species level using MALDI-TOF MS and/or DNA sequencing, together with macroscopic/microscopic observations of cell morphology, galactomannan, and 1,3-β-D glucan tests (data not shown), chest CT imaging, and/or bronchoscopic analyses (Figure 1 and Supplementary Figure 1). The duplicate isolates from the same patients with similar susceptibility profiles were removed from the analysis. There were 20 patients with proven IA (12.82%), 135 patients with probable IA (i.e., probable invasive pulmonary aspergillosis, 86.54%), and one patient with possible IA (0.64%). Among those with probable invasive pulmonary aspergillosis, 30 cases were available with typical pulmonary aspergillosis CT images, including 14 of inflammatory/pulmonary interstitial/well-circumscribed lesions, 4 of fibrous foci, and 12 of distinctive characteristics (6 cases with patchy nodules, 4 cases with cavities, and 2 cases with air crescent signs). The remaining cases were examined *via* bronchoscopy or microscopic observation of *Aspergillus* elements, and they presented typical clinical features as described earlier (Supplementary Figure 2). Additionally, 14 cases were tested serologically positive in galactomannan assays.

**Figure 2:**
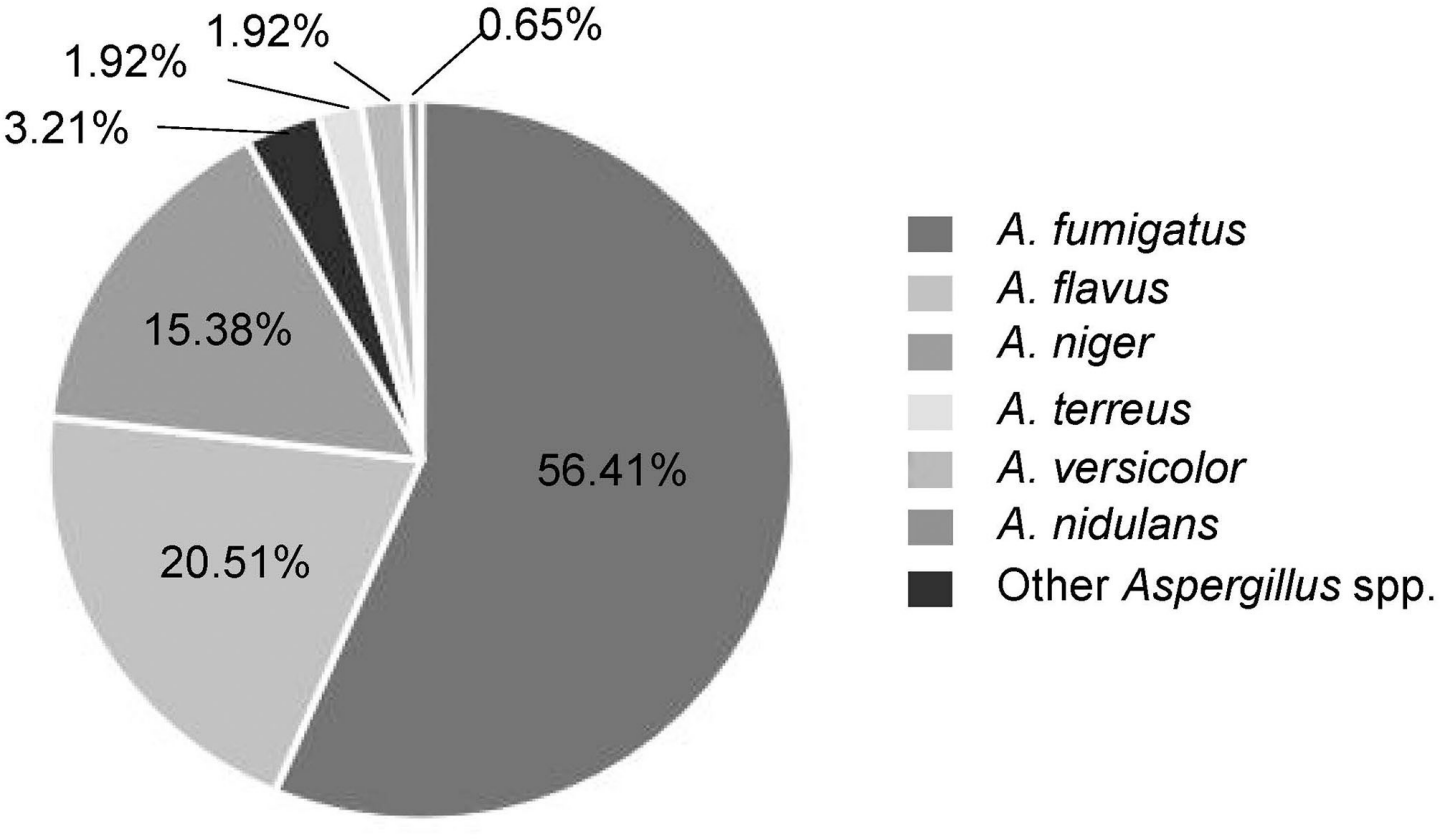
The species distribution of 156 *Aspergillus* isolates in the present study.

Among these *Aspergillus* spp., *A. fumigatus* was the predominant species isolated from patients with invasive *Aspergillus* infections which accounted for 56.41%, followed by *A. flavus* (20.51%), *A. niger* (15.38%), *A. terreus* (1.92%), *A. versicolor* (1.92%), and others (Figure 2). The specimens were of different sources. Most of *Aspergillus* isolates (*n* = 135, 86.54%) were recovered from respiratory specimens, including sputum from deep lower respiratory tracts (*n* = 123, 78.85%) and BALF (*n* = 12, 7.69%). Other specimen sources included sterile sites (mainly blood, ascites, fine-needle aspirates, *etc*.; *n* = 20, 12.82%) and colorectal content (*n* = 1, 0.64%) (Table 1). In the local region, *A. fumigatus* was the main infectious agent for the respiratory system which accounts for 60% (81/135), followed by *A. flavus* 19.26% (26/135), *A. niger* 14.07% (19/135), and *A. terreus* 2.22% (3/135). However, in non-respiratory systems, non-*A. fumigatus Aspergillus* species were dominant (66.67%, 14/21), including all the *A. versicolor* isolates.

**Table 1:** Clinical characteristics of the patients with invasive *Aspergillu*s infections.

**Characteristics**	**Species**						
	***A. fumigatus n =* 88 (%)**	***A. flavus*** ***n =* 32 (%)**	***A. niger*** ***n =* 24 (%)**	***A. versicolor n =* 3 (%)**	***A. terreus*** ***n =* 3 (%)**	***A. nidulans n =* 1 (%)**	**Others** ***n =* 5 (%)**
**Gender**							
Male	60 (58.82)	19 (18.63)	15 (14.71)	2 (1.96)	2 (1.96)	0 (0.00)	4 (3.92)
Female	28 (51.85)	13 (24.07)	9 (16.66)	1 (1.85)	1 (1.85)	1 (1.85)	1 (1.85)
Age*							
0–20 years old	4 (4.55)	0 (0.00)	1 (4.17)	0 (0.00)	0 (0.00)	0 (0.00)	1 (20.00)
21–40 years old	11 (12.50)	6 (18.75)	2 (8.33)	1 (33.33)	1 (33.33)	0 (0.00)	0 (0.00)
41–60 years old	27 (30.68)	8 (25.00)	9 (37.50)	1 (33.33)	2 (66.67)	0 (0.00)	0 (0.00)
61–80 years old	42 (47.72)	15 (46.87)	11 (45.83)	1 (33.33)	0 (0.00)	1 (100.00)	3 (60.00)
>80 years old	4 (4.55)	3 (9.38)	1 (4.17)	0 (0.00)	0 (0.00)	0 (0.00)	1 (20.00)
**Specimen type**							
Sputum	71 (80.68)	25 (78.13)	18 (75.00)	0 (0.00)	3 (100.00)	1 (100.00)	5 (100.00)
BALF	10 (11.36)	1 (3.12)	1 (4.17)	0 (0.00)	0 (0.00)	0 (0.00)	0 (0.00)
Sterile sites	7 (7.95)	6 (18.75)	4 (16.67)	3 (100.00)	0 (0.00)	0 (0.00)	0 (0.00)
Colorectal content	0 (0.00)	0 (0.00)	1 (4.17)	0 (0.00)	0 (0.00)	0 (0.00)	0 (0.00)
**Diagnostic criteria**							
Proven IA	7 (7.95)	6 (18.75)	4 (16.67)	3 (100.00)	0 (0.00)	0 (0.00)	0 (0.00)
Probable IA	81 (92.04)	26 (81.25)	19 (79.17)	0 (0.00)	3 (100.00)	1 (100.00)	5 (100.00)
Possible IA	0 (0.00)	0 (0.00)	1 (4.16)	0 (0.00)	0 (0.00)	0 (0.00)	0 (0.00)
**Underlying disease** ^†^							
Pulmonary disease^#^	30 (34.09)	9 (28.13)	6 (25.00)	0 (0.00)	2 (66.67)	1 (100.00)	3 (60.00)
Hematologic/oncologic disease	10 (11.36)	3 (9.38)	4 (16.66)	2 (66.67)	0 (0.00)	0 (0.00)	2 (40.00)
Neurologic disease	9 (10.23)	1 (3.12)	1 (4.17)	0 (0.00)	0 (0.00)	0 (0.00)	0 (0.00)
Solid organ transplantation	7 (7.95)	4 (12.50)	1 (4.17)	1 (33.33)	0 (0.00)	0 (0.00)	0 (0.00)
Autoimmune disease	6 (6.82)	2 (6.25)	2 (8.33)	0 (0.00)	0 (0.00)	0 (0.00)	0 (0.00)
Hepatic disease	6 (6.82)	2 (6.25)	1 (4.17)	0 (0.00)	0 (0.00)	0 (0.00)	0 (0.00)
Cardiac disease	5 (5.68)	2 (6.25)	4 (16.66)	0 (0.00)	0 (0.00)	0 (0.00)	0 (0.00)
Burns	5 (5.68)	2 (6.25)	1 (4.17)	0 (0.00)	1 (33.33)	0 (0.00)	0 (0.00)
Postoperative condition	4 (4.55)	3 (9.38)	3 (12.50)	0 (0.00)	0 (0.00)	0 (0.00)	0 (0.00)
Virus infection	3 (3.41)	1 (3.12)	0 (0.00)	0 (0.00)	0 (0.00)	0 (0.00)	0 (0.00)
Internal disease	2 (2.27)	1 (3.12)	1 (4.17)	0 (0.00)	0 (0.00)	0 (0.00)	0 (0.00)
Kidney disease	1 (1.14)	2 (6.25)	0 (0.00)	0 (0.00)	0 (0.00)	0 (0.00)	0 (0.00)

* *The IA incidence was correlated with patients' age (except over 80 years old, P <0.001).*

† *It mainly included pulmonary/bronchial inflammation, COPD or tuberculosis; acute lymphoblastic leukemia/myelodysplastic syndrome, solid tumors from lung or gastrointestinal/urological tracts; craniocerebral injury/hemorrhage; kidney/liver transplantation, etc.*

# *The IA incidence was correlated with pulmonary diseases (P = 0.007)*.

**Figure 3:**
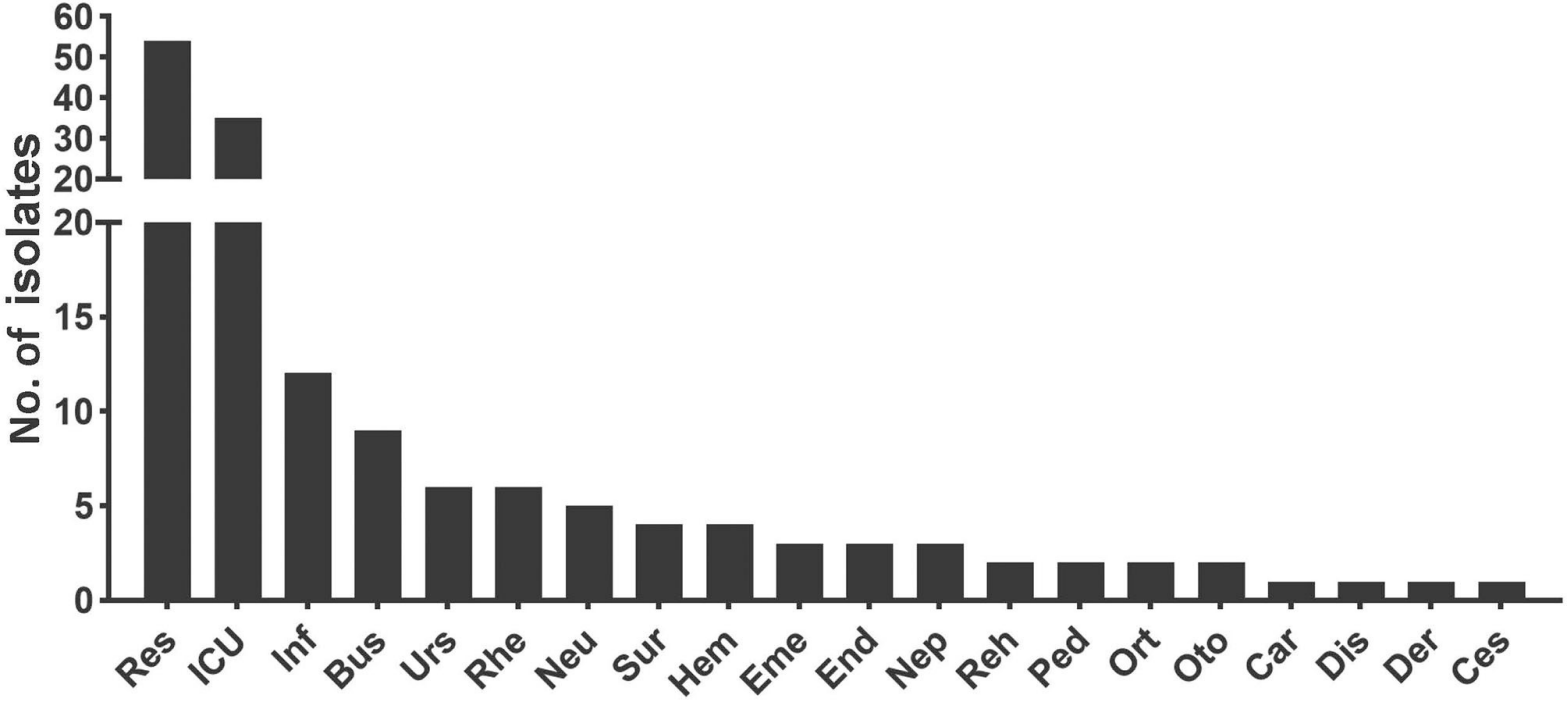
The department distribution of patients infected with invasive *Aspergillus* isolates. Res, Department of Respiratory Medicine; ICU, Intensive Care Unit; Inf, Infection Unit; Bus, Burns Unit; Urs, Department of Urology; Rhe, Department of Rheumatology and Immunology; Neu, Department of Neurology; Sur, Department of Surgery including organ transplantation, hepatobiliary and pancreatic surgery, gastrointestinal surgery, and joint surgery; Hem, Department of Hematology; Eme, Emergency Room; End, Department of Endocrinology; Nep, Department of Nephrology; Reh, Department of Rehabilitation; Ped, Department of Pediatrics; Ort, Department of Orthopedics; Oto, Department of Otolaryngology; Car, Department of Cardiology; Dis, Department of Gastroenterology; Der, Department of Dermatological Venereology; Ces, Department of Chest Surgery.

For all the *Aspergillus* spp. isolated here, Department of Respiratory Medicine contributed the most (34.62%, 54/156), followed by intensive care unit (ICU; 22.44%, 35/156), infection unit (7.69%, 12/156), and burns unit (5.77%, 9/156) (Figure 3). As listed in Table 1, the male to female sex ratio was 1.89 (102 vs. 54), and the most susceptible age was between 61 and 80 years among patients with IA (with a mean age of 58.39 years). In general, the IA incidence grew with age except for patients over 80 years old (correlation analysis, *P* < 0.001). The most prevalent underlying diseases were pulmonary diseases (including pulmonary/bronchial inflammation, chronic obstructive pulmonary disease (COPD), tuberculosis, lung cancer, among others (32.69%, 51/156; correlation analysis, *P* = 0.007). These diseases were followed by hematological/oncological diseases (13.46%, 21/156), solid organ transplantations (8.33%, 13/156), neurological diseases (7.05%, 11/156), and cardiac diseases (7.05%, 11/156), among others.

### Antifungal Susceptibility Patterns

**Figure 4:**
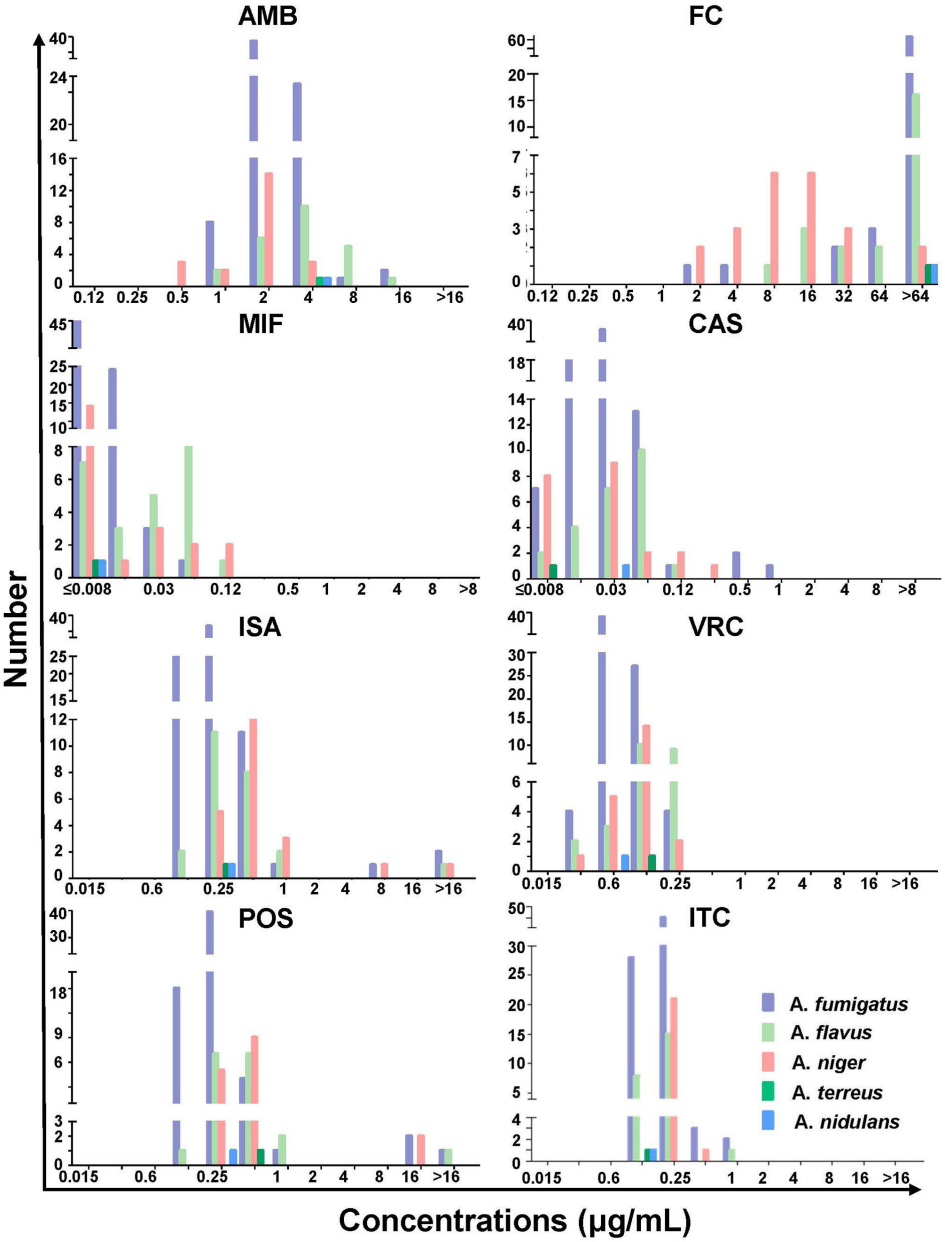
The minimum inhibitory or effective concentration distribution of amphotericin B (AMB), flucytosine (FC), micafungin (MIF), caspofungin (CAS), isavuconazole (ISA), voriconazole (VRC), posaconazole (POS), and itraconazole (ITR) for the indicated *Aspergillus* spp.

Among the 156 *Aspergillus* isolates, apart from those improperly preserved and not applicable for testing, 121 strains were available to undergo antifungal susceptibility assays. The MIC/MEC value, range, and geometric mean (GM) are summarized in Table 2 and are specifically shown in Figure 4. To be noted, except intrinsic resistance, there were 39.73% (29/73) of *A. fumigatus*, 54.17% (13/24) of *A. flavus*, and 13.64% (3/22) of *A. niger* isolates with MICs of more than 2 μg/ml for AMB. The *Aspergillus* spp. showed high MICs (>64 μg/ml) for FC, but not *A. niger* (10.62 μg/ml). On the contrary, they all presented very low MICs for MIF (≤ 0.008 to 0.02 μg/ml) and CAS (≤ 0.008 to 0.03 μg/ml). For azoles, most of the tested isolates had MICs of ≤ 0.5 μg/ml for POS (*n* = 120, 99.17%), ITR (*n* = 117, 96.69%), VRC (*n* = 110, 90.91%), and ISA (*n* = 107, 88.43%). A large proportion of *A. fumigatus* was susceptible to POS (100%), ITR (100%), ISA (95.89%), and VRC (95.89%), among which POS presented the lowest MICs (0.09 μg/ml). The *A. flavus* also displayed a high *in vitro* sensitivity to azoles, albeit 8.33% of resistance to ISA and VRC. Nearly all the other *Aspergillus* spp. (i.e., *A. niger, A. terreus*, and *A. nidulans*) harbored sensitive phenotypes to all azole drugs tested.

**Table 2:** Antifungal susceptibility patterns and characteristics in the five common *Aspergillus* species.

**Species (*n =* 121)**	**MIC or MEC (μg/mL)** *								
		**AMB**	**FC**	**MIF**	**CAS**	**ISA**	**VRC**	**POS**	**ITR**
*A. fumigatus* (*n =* 73)	GM	2.61	>64.00	0.007	0.03	0.27	0.28	0.09	0.22
	MIC_50_	2.00	>64.00	≤ 0.008	0.03	0.25	0.25	0.12	0.25
	MIC_90_	4.00	>64.00	0.02	0.06	0.50	0.50	0.12	0.50
	Mode	2.00	>64.00	≤ 0.008	0.03	0.25	0.25	0.06	0.25
	Range	1.00–16.00	2.00–>64.00	≤ 0.008–0.06	≤ 0.008–0.50	0.12–>16.00	0.12–>16.00	0.06–0.25	0.12–1.00
*A. flavus* (*n =* 24)	GM	2.91	>64.00	0.02	0.03	0.47	0.50	0.14	0.22
	MIC_50_	4.00	>64.00	0.03	0.03	0.25	0.25	0.12	0.25
	MIC_90_	8.00	>64.00	0.06	0.06	1.00	1.00	0.25	0.25
	Mode	4.00	>64.00	≤ 0.008/0.06	0.06	0.25	0.25	0.12	0.25
	Range	1.00–16.00	8.00–>64.00	≤ 0.008–0.12	≤ 0.008–0.12	0.12–>16.00	0.12–>16.00	0.03–0.25	0.121.00
*A. niger* (*n =* 22)	GM	1.66	10.62	0.01	0.02	0.66	0.59	0.13	0.32
	MIC_50_	2.00	8.00	≤ 0.008	0.03	0.50	0.50	0.12	0.25
	MIC_90_	4.00	32.00	0.12	0.12	1.00	1.00	0.12	0.25
	Mode	2.00	8.00/16.00	≤ 0.008	0.03	0.50	0.50	0.25	0.25
	Range	0.50–4.00	2.00–>64.00	≤ 0.008–0.12	≤ 0.008–0.25	0.25–>16.00	0.25–>16.00	0.06–2.00	0.12–>16.00
*A. terreus* (*n =* 1)	MIC	4.00	>64.00	≤ 0.008	≤ 0.008	0.25	0.50	0.12	0.12
*A. nidulans* (*n =* 1)	MIC	4.00	>64.00	≤ 0.008	0.03	0.25	0.25	0.06	0.12

* *MIC, minimum inhibitory concentration; MEC, minimum effective concentration; GM, geometric mean. Mode, values with a clear concentration of trend points in the statistical distribution. Range, from minimum and maximum values of inhibitory concentration*.

**Figure 5:**
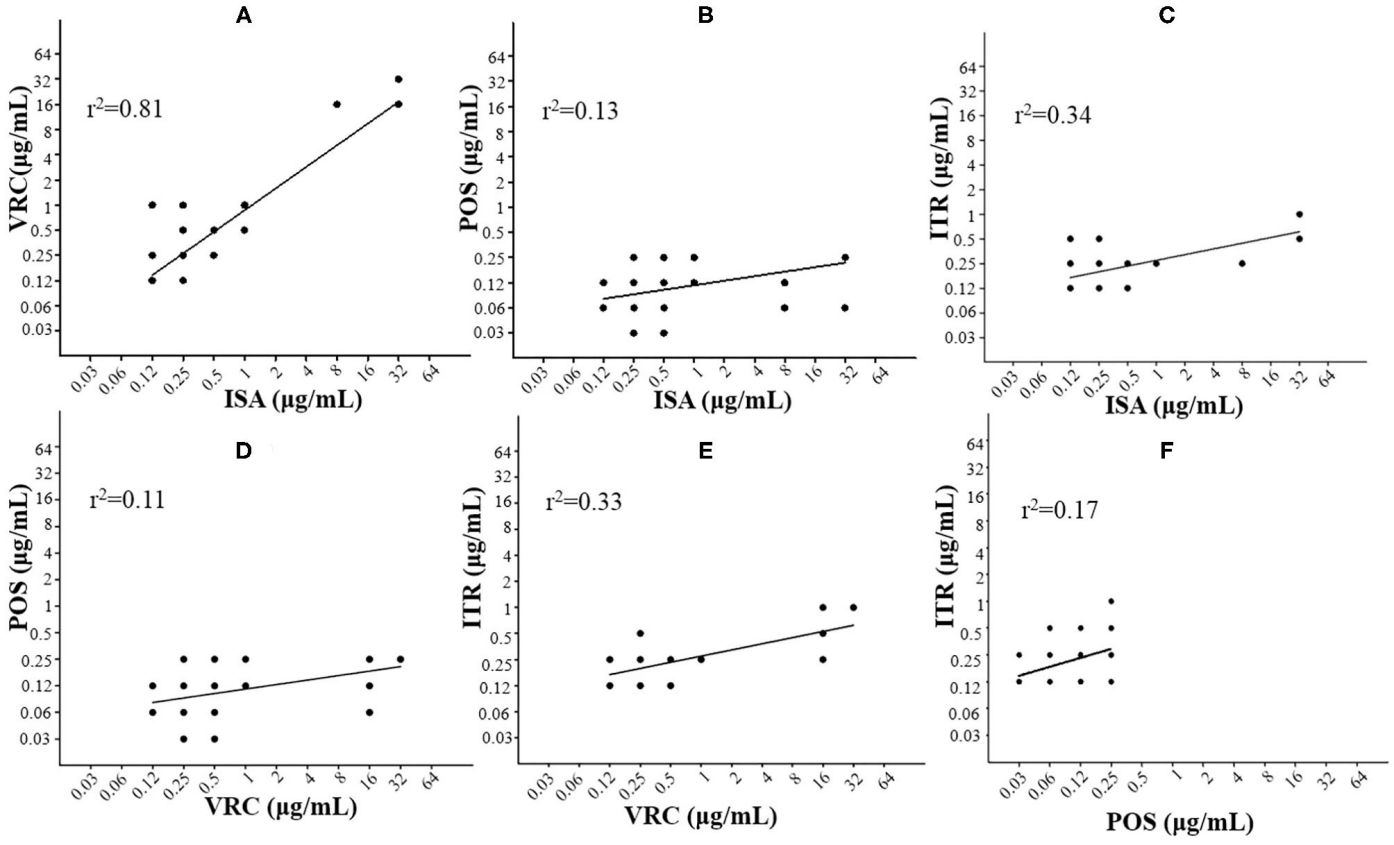
Correlation analyses with scatter plots to compare multiple antifungal minimum inhibitory concentrations (MICs) as indicated. The significant correlation of ISA and VRC (R^2^ = 0.81) was shown for *Aspergillus* spp. A strong correlation suggests considerable cross-resistance. **(A)** ISA vs VRC; **(B)** ISA vs POS; **(C)** ISA vs ITR; **(D)** VRC vs POS; **(E)** VRC vs ITR; and **(F)** POS vs ITR.

It is worth noting that three *A. fumigatus*, two *A. flavus*, and two *A. niger* was screened out to be azole-resistant according to the antifungal susceptibility data and epidemiological cutoff values (ECVs). The relevance of MICs between different azoles was assessed through correlation analysis using scatter plots, which was able to indicate a considerable cross-resistance if there was a significant MIC correlation between two azoles. As shown in Figure 5, a significant MIC correlation was identified between ISA and VRC (Pearson correlation coefficient was 0.81, *P* < 0.0001).

### Gene Mutations of *Cyp51A* Associated With Azole Resistance in *A. fumigatus*

To further investigate a possible mechanism of azole resistance in *A. fumigatus*, all three were azole-resistant, and typically azole-sensitive *A. fumigatus* isolates were recovered for the purpose. Both the promoter fragment and coding region in the *cyp51A* gene were determined by PCR and sequencing. The full-lengths and nucleotide sequences of the promoter and coding region of *cyp51A* in *A. fumigatus* (GenBank Registration number: AF338659) were used as reference sequences, respectively. As summarized in Table 3, all the azole-resistant *A. fumigatus* possessed a TR46 repeat insertion (TCT AGA ATC ACG CGG TCC GGA TGT GTG CTG AGC CGA ATG AAA GTT G) in the 5'-end upstream of *cyp51A* (GenBank accession number: OL396581). Meanwhile, the sequencing analysis spotted 2 single missense mutations (TAT to TTT and ACC to GCC) in all the azole-resistant *A. fumigatus* isolates, leading to amino acid substitutions of Y121F and T289A, respectively. In addition, almost all the azole-resistant and typically sensitive *A. fumigatus* were identified with 5 missense mutations (F46Y, M172V, N248T, D255E, and E427K) at the amino acid level (GenBank accession number: OL388442 and OL388443).

**Table 3:** Mutations of *cyp51A* gene or its coding protein and cross-resistance patterns in azole-resistant and typical wild-type *Aspergillus fumigatus*.

*A. fumigatus* isolates*	***cyp51A*** **mutations**^**†**^								**MIC (μg/mL)/EUCAST**			
	**TR_**46**_**	**F46Y**	**Y121F**	**M172V**	**N248T**	**D255E**	**T289A**	**E427K**	**ISA**	**VRC**	**POS**	**ITR**
R100001	Y	Y	Y	Y	Y	Y	Y	Y	>16.00	>16.00	0.25	1.00
R100002	Y	Y	Y	Y	Y	Y	Y	Y	>16.00	>16.00	0.25	1.00
R100003	N	Y	N	Y	N	N	N	N	8.00	16.00	0.06	0.25
Azole-sensitive	N	Y	N	Y	Y	Y	N	Y	0.12	0.12	0.06	0.12

* *R, azole-resistant.*

† *Y, the presence of the indicated mutation. N, the absence of the indicated mutation*.

## Discussion

Currently, more than 30 *Aspergillus* spp. have been confirmed to correlate with IA, the most common ones are *A. fumigatus, A. flavus, A. niger, A. terreus*, and *A. nidulans* (21). The prevalence of IA and its associated fungal spectrum widely vary from country to country and even among different regions within a country, due to infection sites, age, climate, geographic conditions, agricultural activities, and others factors (22).

In China, *A. fumigatus* was the most commonly isolated *Aspergillus* spp., followed by *A. flavus, A. niger*, and others (23–25). In addition, the incidence of IA caused by non-*A. fumigatus Aspergillus* species has increased in China in recent years. Wang et al. reported that the incidence of invasive pulmonary aspergillosis (IPA) caused by *A. flavus* was greater than that by *A. fumigatus* in patients with hepatitis B virus-related liver failures (26). Li et al. also found an increase in the number of IA cases with *A. niger* and *A. tuberculosis* in clinical and environmental samples from China (27). In this study, more than half of the strains we identified were *A. fumigatus*, then mainly followed by *A. flavus* and *A. niger*. Previous studies revealed that IA was identified primarily in respiratory and urinary tracts (28, 29). In the present study, the clinical specimens of invasive *Aspergillus* infections were mainly from deep lower respiratory tracts out of various clinical departments/units, especially including the Department of Respiratory Medicine, infection unit, ICU, and burns unit. Patients from these units often experience serious illnesses and long-term hospitalization and are subject to extensive use of antibiotics, hormones, immunosuppressants, and/or invasive operations. Thus, these patients usually with a high rate of IA should draw special attention during clinical treatments.

Many patients with IA in our study were men over 40 years of age, similar to previous studies (30). IA is often involved in patients with preexisting pulmonary disorders, such as COPD, active or previous tuberculosis, and previously treated lung cancers (31, 32). In the present study, the most common underlying systemic condition was pulmonary diseases, mainly including pulmonary/bronchial inflammation and COPD. Thus, pulmonary diseases were identified as important risk factors for invasive *Aspergillus* infections.

In clinical settings, antifungal susceptibility patterns are constantly compelling concerns. The antifungal agents are generally categorized into different groups including polyenes, fluoropyrimidine analogs, azoles, echinocandins, etc. (33). This study suggests that the AMB susceptibility to *Aspergillus* spp. in local China would be similar to other studies (34–36), which should be under close surveillance. Resistance to AMB is usually caused by decreased ergosterol or changed lipids of the target in plasma membrane due to alterations of ergosterol biosynthetic pathways which lead to decreased abilities to binding AMB (37), germination of conidia by UV irradiation (38), and mutations of genes encoding sphingolipids FEN1 and SUR4 (39). In our study, 38.84% of *Aspergillus* spp. were resistant to AMB, for which the specific resistance mechanisms need further investigations. The efficacy of FC in treating IA is controversial (40, 41). Our findings demonstrated that *Aspergillus* spp. showed high MIC values to FC, indicating that administration of FC may be an unwise treatment option.

Over the past decades, the frequency of azole resistance has dramatically increased globally (8–10). Resistance of *Aspergillus* spp. to azoles is growing and has become a global health problem. New resistance mechanisms continue to emerge and jeopardize the role of azoles in the treatment of aspergillosis, directly affecting clinical outcomes. The data from the global antifungal surveillance program showed that 5.79% of *A. fumigatus* elevated MICs for one or more azoles (21). The reported frequency of azole resistance differs remarkably between countries/regions, and the antifungal resistance makes the diagnosis and treatment of aspergillosis more complicated (42). The recent *Aspergillus* guidelines from the European Society for Clinical Microbiology and Infectious Diseases (ESCMID), the European Confederation of Medical Mycology (ECMM), and the European Respiratory Society (ERS) recommend that first-line treatment with liposomal AMB or azole plus echinocandins should be considered if the resistance rate exceeds 10% (42). Due to the lack of clinical breakpoints or epidemiological thresholds, the CLSI epidemiological thresholds currently established in *A. fumigatus* species complex are carefully referenced in the present study. Our local rate of resistance to azoles in *Aspergillus* spp. was 5.79% (6/121), with 4.11% (3/73) in *A. fumigatus*, similar to the report by Deng et al. (8) that azole-resistant *A. fumigatus* was prevalent in East and Southeast China (8, 23, 34).

Azoles are able to target the ergosterol biosynthesis process by inhibiting the fungal cytochrome P450-dependent enzyme wool sterol 14-α-demethylase, leading to altered cell membrane function and cell death. The reliable safety and therapeutic efficacy of azoles make them considerately suitable for prophylactic and empirical treatment to severely immunosuppressed patients (43). Notably, ISA, a broad-spectrum azole, showed substantial bioavailability (about 98%) and potent activities in animal models of IA, trichomoniasis, invasive candidiasis, and cryptococcosis (44). Though ISA has not been approved for clinical use in China, *A. fumigatus* isolates presented reduced *in vitro* activities to it, likely due to the cross-resistance of the azole family (45). The azole resistance in *Aspergillus* spp. is associated with multiple distinct adaptive strategies (46–48), among which overexpression and/or open reading frame (ORF) mutation of *cyp51A* gene can alter the binding affinity of azoles to the enzyme, wool sterol 14-α-demethylase (48). In 2011, ARTEMIS DISK Global Antifungal Surveillance Project first reported that TR34/L98H mutation could be found in *A. fumigatus* collected in China (21). Subsequently, Liu et al. declared that the TR34/L98H mutation was the predominant mutation in China and also was quite common in Europe and some other Asian countries (25, 49). Further studies from China have identified more mutations in *A. fumigatus* including TR34/L98H/S297T/F495I, TR34/L98H, G432A, M220I, and TR46/Y121F/T289A (21, 25, 43, 50). To date, azole-resistant *A. fumigatus* has spread mainly in southeastern and northern China (51). In this study, we reported that TR46/Y121F/T289A mutations were found in two-thirds of azole-resistant *A. fumigatus* isolates and they exhibited very high MIC values to ISA and VRC. We seldom observed mutant strains with TR34/L98H mutation and/or TR53, thus the TR46/Y121F/T289A mutation pattern is likely the dominant phenotype in the local region. The extensive use of sterol demethylation inhibitors (DMIs) in agriculture may contribute to the increasing emergence of azole-resistant *A. fumigatus*. The presence of tandem repeats upstream of the 14-α-demethylase gene is an important mechanism found in phytopathogenic mycorrhizal fungi that can develop resistance to azoles through DMI fungicides exposure and function as a transcriptional enhancer, allowing significant overexpression of *cyp51A* gene (52–55). Invasive *Aspergillus* infections in immuno-competent hosts are uncommon, though an immuno-competent adult in our study was infected with *A. fumigatus* after a drowning event, and the isolated strains showed azole resistance in an *in vitro* drug susceptibility test, supporting the possibility that the resistant strains might be of environmental origin (56). Other mutation-harboring *A. fumigatus* strains found in our research, including F46Y, M172V, N248T, D255E, and/or E427K, are scarcely related to azole resistance (12, 57). In addition, in one azole-resistant *A. fumigatus* isolate, none of TR46/Y121F/T289A mutations were found, suggesting that other mechanisms may be involved and need to be further investigated (8, 58).

## Conclusions

In summary, this study reported the *Aspergillus* species distribution and antifungal sensitivities, and clinical characteristics and risk factors of patients with IA of a local region in central China. The *A. fumigatus* and *A. flavus* were still the major pathogens for invasive *Aspergillus* infections here. The vast majority of *Aspergillus* spp. exhibited good susceptibility to almost all the antifungals commonly used in clinics. The findings implied that pulmonary diseases and age are likely considered as the main risk factors for such infections. The polymorphism of the *cyp51A* gene in *A. fumigatus* may be closely associated with azole resistance, contributing to treatment failures in patients with IA. Their important clinical implications emphasize the need for antifungal susceptibility surveillance and screening out mutations in resistance-linked genes.

## Data Availability Statement

The data presented in the study are available in the Genbank repository, accession number OL396581, OL388442 and OL388443s.

## Ethics Statement

The studies involving human participants were reviewed and approved by the Life Ethics Committee of Anhui Medical University. Written informed consent to participate in this study was provided by the participants' legal guardian/next of kin.

## Author Contributions

YW, MZ, and YX conceived and designed the experiments, contributed to the interpretation of results, and assisted in writing the manuscript. YW, LZho, MZ, and YX designed the research protocol and performed the experiments. YW, LZha, LZho, MZ, and YX performed data acquisition and analysis. All authors read and approved the final manuscript.

## Funding

This study was supported by the Foundation of the Anhui Science and Technology Department (201904a07020049) (YX). The funders had no role in the study design, data collection and analysis, decision to publish, or preparation of the manuscript.

## Conflict of Interest

The authors declare that the research was conducted in the absence of any commercial or financial relationships that could be construed as a potential conflict of interest.

## Publisher's Note

All claims expressed in this article are solely those of the authors and do not necessarily represent those of their affiliated organizations, or those of the publisher, the editors and the reviewers. Any product that may be evaluated in this article, or claim that may be made by its manufacturer, is not guaranteed or endorsed by the publisher.

## Abbreviations

IA: invasive aspergillosis
CPA: chronic pulmonary aspergillosis
IFD: invasive fungal disease
EORTC/MSG: the European Organization for the Research and Treatment of Cancer/Mycoses Study Group
BALF: bronchoalveolar lavage fluid
MALDI-TOF MS: matrix-assisted laser desorption ionization-time of flight mass spectrometry
SYO: Sensititre YeastOne
MIC: minimum inhibitory concentration
MEC: minimum effective concentration
GM: geometric mean
MIC_50_: MIC/MEC at which 50% of the isolates tested were inhibited
MIC_90_: MIC/MEC at which 90% of the isolates tested were inhibited
AMB: amphotericin B
FC: flucytosine
MIF: micafungin
CAS: caspofungin
ISA: isavuconazole
VRC: voriconazole
POS: posaconazole
ITR: itraconazole
EUCAST: the European Committee on Antibiotic Susceptibility Testing
CLSI: the Clinical and Laboratory Standards Institute
PCR: polymerase chain reaction
ICU: intensive care unit
COPD: chronic obstructive pulmonary disease
IPA: invasive pulmonary aspergillosis
ESCMID: the European Society for Clinical Microbiology and Infectious Diseases
ECMM: the European Confederation of Medical Mycology
ERS: the European Respiratory Society
ORF: open reading frame.
