# Supplementary material for: Epidemiology, Drug Susceptibility, and Clinical Risk Factors in Patients With Invasive Aspergillosis
Source: Front Public Health. 2022 Apr 15;10:835092. doi: 10.3389/fpubh.2022.835092 (PMC9051236; doi:10.3389/fpubh.2022.835092)
Supplement: Supplementary file 1 [file Data_Sheet_1.DOCX]

**Supplementary Materials for “Epidemiology, Drug Susceptibility and Clinical Risk Factors in Patients with** **Invasive Aspergillosis”**


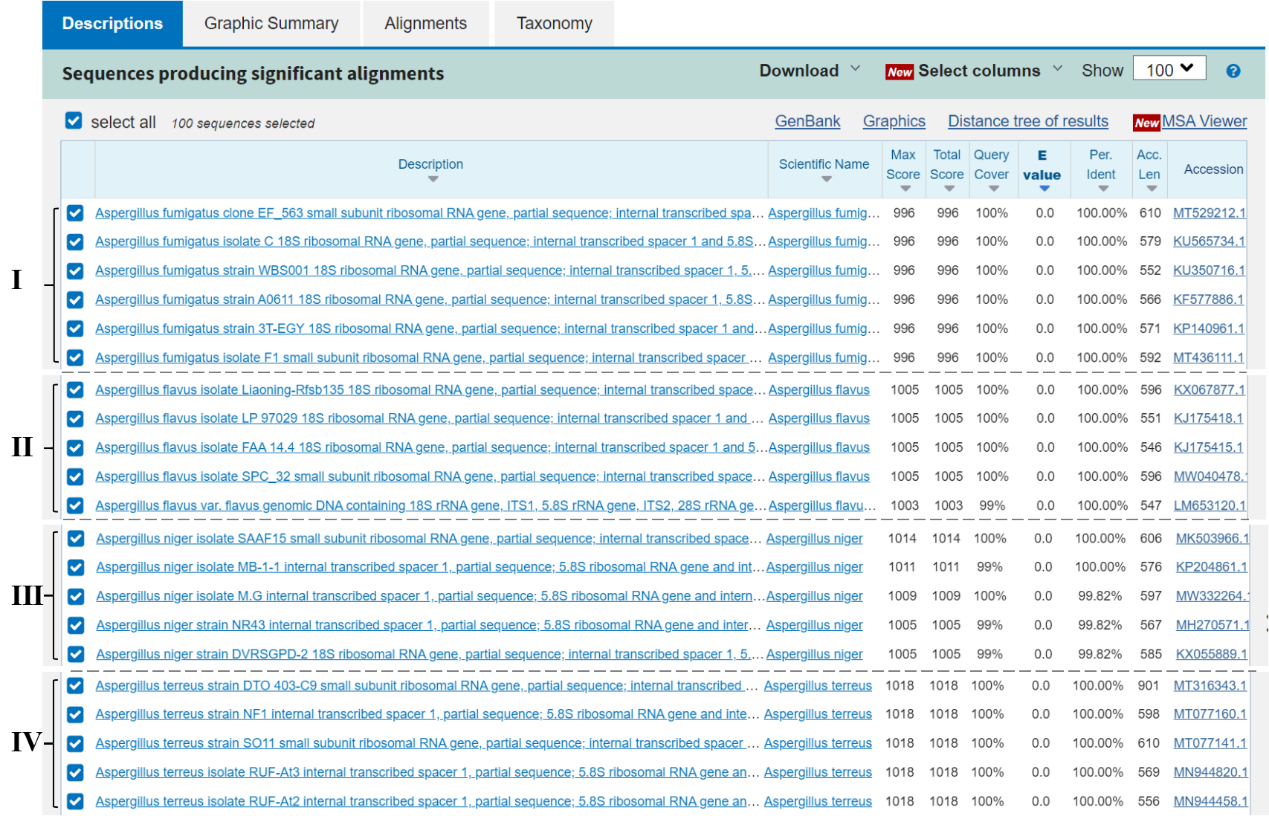


**Figure S1.** Representative analysis of the internal transcribed spacer (ITS) region nucleotide sequences from *Aspergillus* spp. identified in the present study using BLAST program. I ~ IV: *A. fumigatus*, *A. flavus*, *A. niger*, and *A. terreus*.


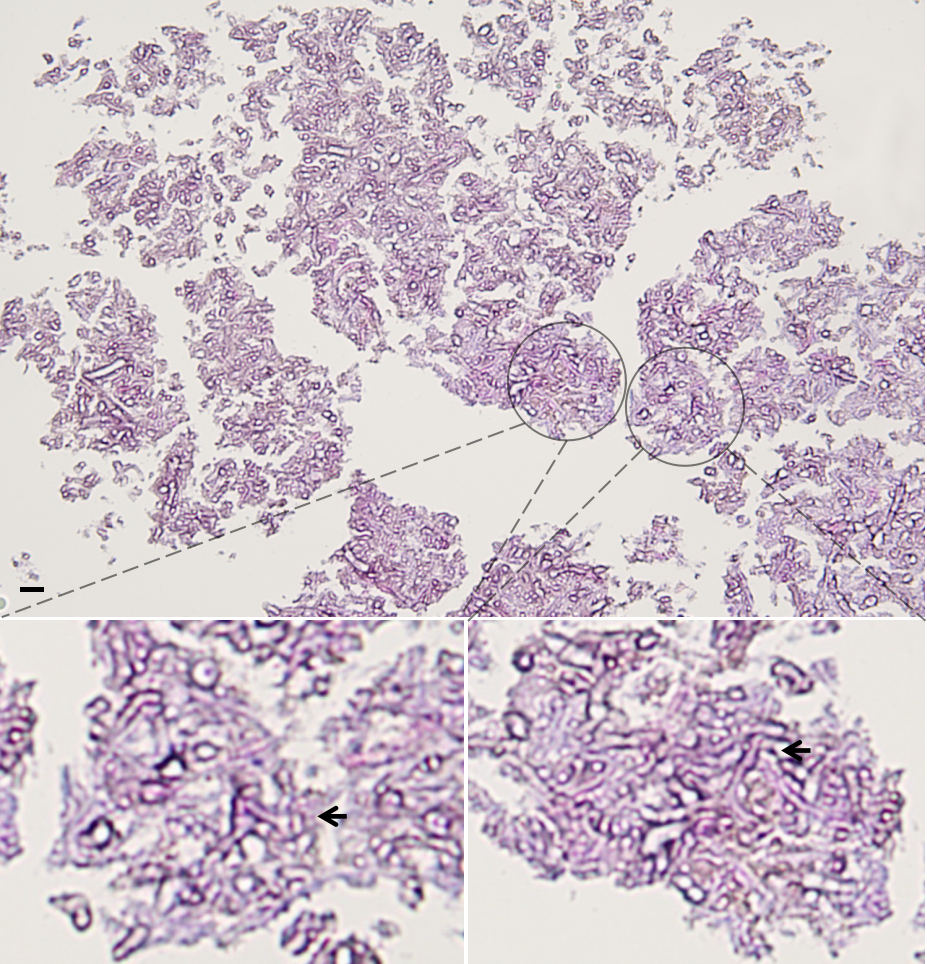


**Figure S2.** Representative HE staining data of direct smear of bronchoscopic specimens. Blank arrows, mycelia of *Aspergillus fumigatus*. Scale bar: 20 μm.
